# Review of Insulin Resistance in Dilated Cardiomyopathy and Implications for the Pediatric Patient Short Title: Insulin Resistance DCM and Pediatrics

**DOI:** 10.3389/fped.2021.756593

**Published:** 2021-10-28

**Authors:** Daniel Mak, Kaitlin A. Ryan, Joan C. Han

**Affiliations:** ^1^Division of Pediatric Endocrinology, Department of Pediatrics, The University of Tennessee Health Sciences Center, Memphis, TN, United States; ^2^Division of Pediatric Cardiology, Department of Pediatrics, The University of Tennessee Health Sciences Center, Memphis, TN, United States

**Keywords:** insulin resistance, pediatric, antidiabetic medications, dilated cardiomyopathy, SGLT-2 inhibitor

## Abstract

Energy metabolism in the heart is affected during states of dysfunction. Understanding how the heart utilizes substrates in cardiomyopathy may be key to the development of alternative treatment modalities. Myocardial insulin resistance has been proposed as a possible barrier to effective glucose metabolism in the heart. Extensive literature on the topic in adult individuals exists; however, review in the pediatric population is sparse. The pathophysiology of disease in children and adolescents is unique. The aim of this paper is to review the current knowledge on insulin resistance in dilated cardiomyopathy while also filling the gap when considering care in the pediatric population.

## Introduction

Children with cardiomyopathy are a vulnerable population and understanding the factors that contribute to cardiac dysfunction are of great importance. The annual incidence of pediatric cardiomyopathy in the United States is ~1.13 cases per 100,000 children aged 18 years or younger which is similar to other major population studies (1–3). Among cardiomyopathies seen in children, dilated cardiomyopathy (DCM) is the most common. From the Pediatric Cardiomyopathy Registry, the annual incidence of DCM in patients under 18 years old was 0.57 cases per 100,000 per year (4). Most individuals have idiopathic disease, thus limiting potential disease-specific treatments (4). While rare, pediatric cardiomyopathy has high morbidity and mortality. Pediatric cardiomyopathy is the leading cause of heart transplantation after 1 year of age (5). It also has a high health-care burden that is significantly greater in children than compared to adults. In an analysis of cardiomyopathy and heart failure-related hospitalizations in the United States, pediatric patients were hospitalized for significantly longer durations with overall greater mortality resulting in higher costs than adult patients (6). Thus, other modalities to improve care of cardiomyopathy in children are needed. At the biochemical level, understanding energy utilization by cardiomyocytes during stress may provide insight into the progression of cardiomyopathy. There is a large body of literature that describes insulin resistance in adults with cardiomyopathy. Insulin resistance is classically defined as the body's impaired ability to respond to the actions of the hormone insulin on glucose homeostasis (7, 8). It is well known that insulin resistance is an intrinsic part of type 2 diabetes which in turn is a risk factor for coronary artery disease and atherosclerosis leading to cardiovascular dysfunction (9, 10). It is also known with diabetes that there can be impairment of the cardiac muscle itself, which is termed diabetic cardiomyopathy. Interestingly, insulin resistance is also present in individuals with cardiomyopathy without diabetes, which suggests a potential bi-directional relationship between cardiomyopathy and insulin resistance. The purpose of this review is to discuss the interplay between DCM and insulin resistance and its applicability to the pediatric patient.

## Energy Homeostasis in the Heart

In healthy hearts, during the fasting state, energy is primarily derived from the oxidation of free fatty acids (FFA) (60–70%), followed by glucose (~20%) and lactate (~10%). In the post-prandial state, glucose is the preferred substrate (60–70%) (11, 12). It has been shown that during increased cardiac workload (physical exertion or stress testing), the heart can exhibit metabolic flexibility and switch predominately to glucose oxidation (13–15). Since glucose is a more efficient substrate, this ability confers an adaptive advantage. Of interest is whether this metabolic flexibility is preserved in cardiac disease states such as DCM. Dávila-Román et al. (16) used positron emission tomography (PET) to demonstrate that in adults with idiopathic dilated cardiomyopathy (IDCM) there was greater myocardial glucose uptake and utilization while having decreased fatty acid uptake and utilization in the fasting state (16). Neglia et al. (17) also showed that in IDCM there was a baseline preference of glucose utilization over fatty acids in the resting state compared to control subjects however, during physical exertion further adaptation was reduced. During atrial pacing, there was an increase in glucose uptake in control subjects however this was not seen in subjects with IDCM and instead lactate production increased (17). These results suggested a maladaptive response to substrate utilization in subjects with IDCM during physical stress. Interestingly, Tuunanen et al. (18) found that in individuals with IDCM FFA metabolism was reduced at baseline compared to controls but with worsening left ventricular (LV) function myocardial FFA uptake and oxidation increased. Insulin resistance [using the homeostasis model assessment index (HOMA index)] was found to be positively correlated with FFA oxidation (18).

The healthy heart has metabolic flexibility to vary substrate utilization based on the cardiac workload. In IDCM, that metabolic flexibility may be decreased or lost secondary to insulin resistance.

## Insulin Signaling in the Heart

The activation of insulin signaling pathways in cardiac muscle is similar to that in striated muscle (12, 19). The function of insulin is to promote translocation of the insulin-sensitive GLUT4 transporter from the cytosol to the plasma membrane, which in turn facilitates glucose utilization by the cell. Binding of insulin to its receptor activates a cascade of intracellular signaling pathways, the mitogenic-activated protein kinase (MAPK) pathway and the metabolic phosphatidylinositol 3-kinase (PI3K) pathway. The MAPK pathway is involved in cell growth and differentiation while activation of the PI3K pathway regulates nutrient metabolism. Activation of PI3K pathway leads to activation of Akt, also known as protein kinase B (PKB). Akt/PKB plays an essential role in translocation of the GLUT4 transporter to the cell membrane (20). In the heart, the most common glucose transport isoforms are GLUT1 and GLUT4. Other less common isoforms also exist (21). GLUT1 is predominately expressed in the developing embryonic heart but then declines postnatally. In one animal model, inducing LVH by aortic banding, it was found that basal glucose uptake increased and was associated with increased GLUT1 levels. Additionally, overall total GLUT4 expression was reduced but the proportion in the cellular membrane was increased (22). The mechanisms of intracellular insulin signaling in humans with cardiac dysfunction is complex and incompletely understood. Cook et al. (23) showed that in patients with LV dysfunction, there was an increase in cardiac PI3K activity and increase in sarcolemmal GLUT4 (23). Conversely, another study by Chokshi et al. (24) demonstrated in advanced heart failure (in need of ventricular assist device), there was evidence of reduced activation of the insulin signaling cascade and it correlated with increases in toxic lipid intermediates (24).

In stable DCM, increases in glucose oxidation may be related to increased translocation of GLUT4 to the cell membrane, however with advanced disease there may be reduced activation of the insulin signaling cascade.

## Cardiomyopathy and Heart Failure as an Insulin Resistant State

It is widely known that insulin resistance and diabetes mellitus can lead to cardiomyopathy (25–28). It has also been suggested that cardiac dysfunction seen in IDCM without diabetes is also associated with both whole-body and myocardial insulin resistance (19, 29). In canine models, where DCM was induced, increasing severities of LV dilation and dysfunction were associated with myocardial and whole-body insulin resistance as evidenced by a reduction in both whole-body and myocardial glucose uptake while insulin levels increased significantly (30). In humans, Swan et al. (31) found that adults with congestive heart failure (CHF) had marked insulin resistance as demonstrated by elevated insulin and c-peptide levels during fasting as well as response to glucose load on tolerance test, when compared to controls with similar BMI (31). Witteles et al. (32) showed similar results in patients with IDCM as compared to BMI-matched controls (32).

**Figure 1:**
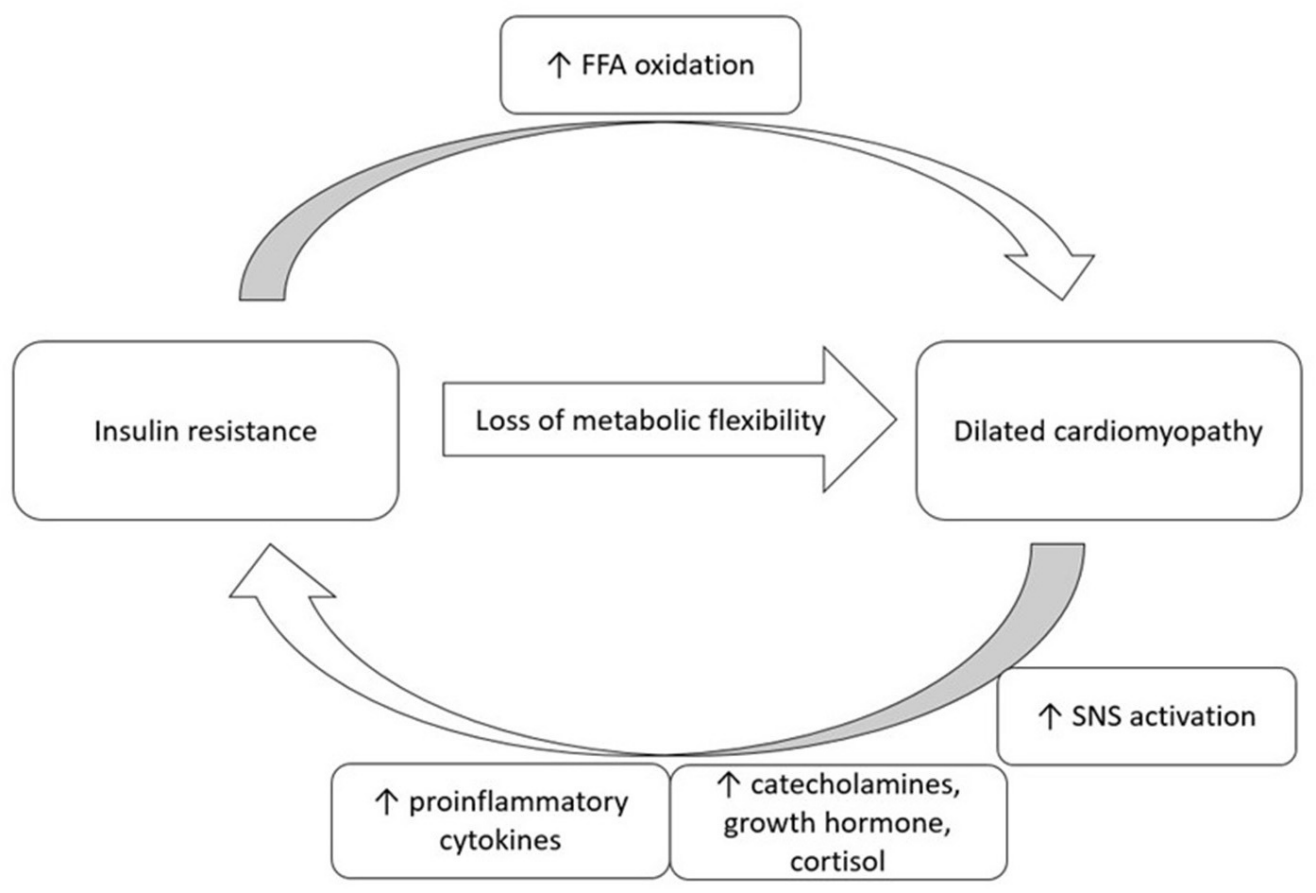
A proposed bi-direction relationship of insulin resistance and dilated cardiomyopathy. FFA, free fatty acid; SNS, sympathetic nervous system.

The pathophysiology of insulin resistance in cardiomyopathy is multifactorial. Cardiac dysfunction leads to increased activation of the sympathetic nervous system (SNS). Increased levels of proinflammatory cytokines, catecholamines, growth hormone, and cortisol are all associated with insulin resistance (see Figure 1) (19). Paolisso et al. (33) demonstrated that insulin resistance was associated with CHF and found associated elevated plasma norepinephrine and tumor necrosis factor-α (TNF-α) concentrations (33). Sakai et al. (34) also showed that individuals with DCM had significantly higher HOMA-IR when compared to individuals with valvular heart disease. There was also an observed trend toward higher TNF-α concentrations in individuals with DCM (34).

There is a potential bi-directional relationship between IDCM and insulin resistance. In states of cardiac dysfunction, increased activation of the SNS and increases in proinflammatory cytokines may lead to insulin resistance.

## Implications for the Pediatric Patient

It is often said that “children are not little adults” and that conditions in pediatric patients are different from their counterparts in adults. The natural history of IDCM in children can be highly variable, including more rapid and severe disease, such that IDCM is the most common indication for heart transplant in children older than 1 year (35). Literature shows that after IDCM diagnosis, 5-year survival is 76% and 10-year survival is 74%. Avoidance of death or transplantation at 5-years is 47% and at 10-years is 42% (4). Moreover, the treatments used in adults are not always as effective in children. One study demonstrated that unlike adults, children with DCM do not undergo adverse cardiac remodeling and thus typical medications that target cardiac remodeling in adults are ineffective in children (36). The Pediatric Randomized Carvedilol Trial in Children with Heart Failure did not find significant improvement in clinical heart failure outcomes with use of carvedilol in children and adolescents with symptomatic systolic heart failure though this medication is commonly used to treat adults with heart failure (37). Interestingly though, while pediatric cardiomyopathy can be an aggressive disease, unlike adults, some children may experience recovery. One large multi-center study showed that ~20% of children after 2 years of diagnosis with IDCM had normalization of cardiac function. Normalization was associated with younger age (<10 years old) and less severe LV dilation at diagnosis (38). Research into specific biomarkers than can predict recovery is important and provides promise for potential therapeutic approaches.

One physiologic difference to highlight in children and adolescents as compared to adults is the onset and progression of pubertal development. Rising growth hormone levels can contribute to insulin resistance. Puberty has been shown to be associated with a decrease in insulin-stimulated glucose metabolism in patients with and without diabetes (39). When elucidated further, it seems that rising growth hormone and not sex steroids influence insulin resistance (39, 40). It is also known that growth hormone is a counter-regulatory hormone to the action of insulin and thus has an influence on insulin sensitivity (41). In one comprehensive study, it was shown that across Tanner stages, the rise and fall in IGF-1 levels corresponded to changes in insulin resistance as measured during an euglycemic hyperinsulinemic clamp study (42). Additionally, in patients born small-for-gestational age treated with growth hormone therapy, long-term treatment was associated with increases in insulin resistance as measured by HOMA-IR and insulinogenic index (43). Thus, children and adolescents have factors that promote insulin resistance, and this may have a compound effect on insulin resistance seen in cardiac dysfunction.

Disease progression in children with IDCM can be more rapid and severe than in adults. Among other factors, physiologically rising growth hormone during puberty can increase insulin resistance.

## Antihyperglycemic Agents And Heart Failure Outcomes

Given the knowledge that insulin resistance may be a contributing factor to worsening cardiac function, there is interest in using medications that can improve insulin sensitivity, glucose homeostasis and in turn possibly also have beneficial cardiac effects. There have been several studies that evaluated the effect of anti-hyperglycemic agent classes on risk for heart failure and other major adverse cardiovascular events (MACE), including risk of cardiovascular death, non-fatal myocardial infarction, and non-fatal stroke in patients with and without diabetes (44, 45). Of the multitude of agents available to adults, only metformin and liraglutide have been approved by the Food and Drug Administration (FDA) for use in the pediatric population with type 2 diabetes. While not approved for use in pediatric age group yet, one agent class, the SGLT-2 inhibitors show promise in both improving glycemic control as well as cardiovascular function.

Metformin is a widely used oral anti-hyperglycemic agent for type 2 diabetes, and randomized clinical trials have shown an association with reduced macrovascular events (46). Metformin exerts its effect by decreasing hepatic glucose production, decreasing intestinal absorption of glucose as well as promoting the translocation of GLUT1 and GLUT4 to the sarcolema to increase peripheral glucose uptake and utilization (47). Bjornstad et al. (48) showed that in adolescents and young adults with type 1 diabetes, use of metformin was associated with improvements in key measures of vascular health including decreases in maximal wall shear stress, pulse wave velocity in the ascending aorta and far-wall diastolic carotid intima-medial thickness (48). Another randomized control trial, EMERALD (Effects of Metformin on Cardiovascular Function in Adolescents with Type 1 diabetes), with results underway, looks to compare change in cardiac function as measured by echocardiogram in patients received metformin compared to placebo (49).

Glucagon-like peptide-1 receptor agonists (GLP-1 RA) act by stimulating glucose-dependent insulin secretion. In murine models of DCM, treatment with exenatide was found to impact glucose homeostasis through improved glucose tolerance, increased myocardial GLUT4 expression and 2-deoxyglucose uptake, and greater cardiac contractility (50). The LIVE study (Liraglutide on Left Ventricular Function in Chronic Heart Failure Patients with and without Type 2 Diabetes) did not show improvement in LV systolic function compared with placebo, in patients with and without type 2 diabetes, and was in fact associated with increased heart rate and more serious cardiac adverse events (51). In other studies of adolescents and adults with and without cardiac dysfunction, increased heart rate was also observed in association with liraglutide treatment (52, 53). There are also several randomized control trials evaluating agents in this drug class in individuals with type 2 diabetes and their risk for MACE (54–58). Results showed that the agents were not inferior to placebo and in some showed a reduction in MACE however none showed any difference in hospitalization for heart failure (54).

The most promising antihyperglycemic agents for improvement in cardiac function are the sodium-glucose co-transporter-2 (SGLT-2) inhibitors, also called gliflozins. SGLT-2 is in the renal proximal tubule to facilitate movement of glucose and sodium. Inhibition of this co-transporter inhibits glucose reabsorption from the kidney, causing glycosuria and thereby lowering blood sugar. There are several studies that examined the effect of SGLT-2 inhibitors on cardiovascular outcomes in patients with type 2 diabetes. Both EMPA-REG OUTCOME (Empalifilozin, Cardiovascular Outcomes, and Mortality in Type 2 Diabetes) and CANVAS (Canagliflozin Cardiovascular Assessment Study) trials found that in individuals with type 2 diabetes, use of SGLT-2 inhibitors was associated with a reduction in death from cardiovascular causes, hospitalization for heart failure, and all-cause mortality when compared with placebo (59–61). Recent studies exploring the role of SGLT-2 inhibitors in patients with HF without diabetes have also shown promising results. The DAPA-HF trial (Dapagliflozin and Prevention of Adverse-Outcomes in Heart Failure) showed that in individuals with and without diabetes, dapagliflozin was clearly superior to placebo at preventing heart failure events and cardiovascular deaths (62, 63). There are several mechanisms proposed for the cardiovascular benefits seen with SGLT-2 inhibitors (64). Possible mechanisms for improved cardiovascular function may be attributable to the effect of SGLT-2 inhibitors on volume status which may result in increased natriuresis, decreased myocardial fibrosis, and increased cardiac contractility (65, 66). However, results from the EMPEROR-reduced trial (Empagliflozin Outcome Trial in Patients with Chronic Heart Failure with Reduced Ejection Fraction) did not show a difference in outcomes in patients with recent volume overload and those without. Thus, it was concluded that increased natriuresis was unlikely to be the predominant factor in the clinical benefits of the medication (67). SGLT-2 inhibitors have also been shown to cause an increase in ketone levels which presumably is due to glucosuria and thus energy deficiency. Another proposed mechanism of how SGLT-2 inhibitors benefit cardiovascular function is through changing the heart's substrate utilization to adapt to ketone oxidation (68). There is emerging literature that supports the proposal of ketone oxidation as an alternative fuel source in the failing heart (69). There may be a role for antihyperglycemic agents in modulating insulin resistance and secondarily on cardiac function. Clinical studies are currently underway in the pediatric population in patients with diabetes mellitus. Future studies will be important in examining the cardioprotective nature of these agents in patients without diabetes.

## Conclusions

The heart undergoes adaptive metabolic changes in times of acute and chronic stress. Understanding cardiac energy homeostasis may be key in unraveling causes of progressive dysfunction in disease states. In cardiomyopathy and heart failure, there is loss of metabolic flexibility. It has been suggested that cardiomyopathy is an insulin resistance state and this limits cardiac metabolic efficiency. Cardiomyopathy in the pediatric population is especially delicate with high morbidity and mortality and is in dire need of alternative therapies. Current research investigating the cardiovascular benefits of anti-hyperglycemic agents is promising, particularly SGLT-2 inhibitors. Soon, medications that benefit cardiac energy homeostasis may add a new class of therapeutics to the arsenal to treat pediatric cardiomyopathy.

## Author Contributions

DM was responsible for the conception and draft of the work. KR and JH were responsible for substantial revision of the work. All authors approved the submitted version and agreed both to be personally accountable for the author's own contributions and to ensure that questions related to the accuracy or integrity of any part of the work, even ones in which the author was not personally involved, are appropriately investigated, resolved, and the resolution documented in the literature.

## Conflict of Interest

The authors declare that the research was conducted in the absence of any commercial or financial relationships that could be construed as a potential conflict of interest.

## Publisher's Note

All claims expressed in this article are solely those of the authors and do not necessarily represent those of their affiliated organizations, or those of the publisher, the editors and the reviewers. Any product that may be evaluated in this article, or claim that may be made by its manufacturer, is not guaranteed or endorsed by the publisher.
